# Supplementary material for: Patterns of Intron Gain and Loss in Fungi
Source: PLoS Biol. 2004 Nov 30;2(12):e422. doi: 10.1371/journal.pbio.0020422 (PMC532390; doi:10.1371/journal.pbio.0020422)
Supplement: Table S1 — Also available at http://genes.mit.edu/NielsenEtAl/. (4.3 MB ZIP). [file pbio.0020422.st001.zip › NielsenEtAl/html/245.html]

AN9408.1.NCU07307.1.MG04118.1.FG05322.1


```
 CLUSTAL W (1.82) Multiple Sequence Alignments - Introns Inserted


Sequence 1: NCU07307.1	2092 aa
Sequence 2: MG04118.1	2242 aa
Sequence 3: FG05322.1	2104 aa
Sequence 4: AN9408.1	2093 aa
Alignment Length: 2244 aa
Number Identitical Residues: 1285 aa
Alignment Score (without introns) 60249


MG04118.1 	MYRVFLPVGTLVLIALGATSANNRMKSYGYVALWPIDTQK0GGCGEPQANGLRWHLDAAF
NCU07307.1	----------------------------------------~-------------------
FG05322.1 	----------------------------------------~-------------------
AN9408.1  	----------------------------------------~-------------------
          	                                                            

MG04118.1 	EDFAWRRSGPQVHSPCRSVDFDVIQGRPSGMEGKNAGVQCVDPSQQN1THRHSELRRRTV
NCU07307.1	-----------------------------------------------~------------
FG05322.1 	-----------------------------------------------~------------
AN9408.1  	-----------------------------------------------~------------
          	                                                            

MG04118.1 	TNHNMYGTGTGPQTGVSTPRSSASLRPLNLTHGSLETSFLIPTNLHFHASQLKDRFSASL
NCU07307.1	----MYGTGTGAQTGVTTPRSSASLRPLTVSHGSLETSFLIPTGLHFHATQLKERFAAIL
FG05322.1 	----MYGAGQGAQTGISTPRSSASLRPLTLSHGSLETSFLIPTNLHFHASQLKDKFVATL
AN9408.1  	----MYGTSTGPQTGINTPRSSQSLRPLILSHGSLEFSFLVPTSLHFHASQLKDTFTASL
          	    ***:. *.***:.***** ***** ::***** ***:**.*****:***: * * *

MG04118.1 	PAPTDELAQDDEPSSVPELVARYMGFVAREVEEEGDDAQGSYEEVLKLLLNEFERVFLRG
NCU07307.1	PAPTDELAQDDEPSSIFELVARYMGFIATEVTQGEDDAQGSYEEVLKIILNEFERAFLRG
FG05322.1 	PEATDELAQDDEPSSVPDLVARYLDLIAHEVDEGEDDDAGSYEEVLKLVLNEFERNFLRG
AN9408.1  	PEPTDELAQDDEPSSVAELVARYIGHVAHEVEEGEDDAHGTNQDVLKLALNEFERAFMRG
          	* .************: :*****:. :* ** :  **  *: ::***: ****** *:**

MG04118.1 	NEVHALAATLPGIDTKKLEVIRSYFATRYVLNRAIKPHASALFRASAEDKAKIYTIFGGQ
NCU07307.1	NDVHALVSTMEGIDDKKLEVIRCYYLARSASNRAIKPYESALFRAAGENAAKIFTIFGGQ
FG05322.1 	NEAHAVAANLPGIESKKLDFIRSYYTARSVCNRPIKPHASALFRAAEDGDAEIYTIFGGQ
AN9408.1  	NDVHAVAATLPGITAKKVLVVEAYYAGRAAAGRPTKPYDSALFRAASDEKARIYSVFGGQ
          	*:.**:.:.: **  **: .:..*:  * . .*. **: ******: :  *.*:::****

MG04118.1 	GNIEEYFEELRELHTTYRVFIEELITTSAELLQNLSKDPSAEKLFSKGLDVMSWLHRPEA
NCU07307.1	GNIEEYFEELRELHHTYPAFVGELITTSAELLQTLSSHPDAEKMYPKGLDVLGWLQSPEA
FG05322.1 	GNIEEYFEELREIFKTYSSFVGDLITRSAELLQTLSKDPKAEKMFPKGLDIMNWLHHEDS
AN9408.1  	GNIEEYFDELREVYNTYTSFVDDLISSSAELLQSLSREPDANKLYPKGLNVMQWLREPDT
          	*******:****:. **  *: :**: ******.** .*.*:*::.***::: **:  ::

MG04118.1 	TPDIDYLVSAPVSFPLIGLVQLAHYEVTCKVLGVPPSALRERFSGATGHSQGIVLAAATA
NCU07307.1	TPDVDYLISAPVSFPLIGLVQLAHYQVTCKVLGVHPGVLRERISGTTGHSQGVVLSAITA
FG05322.1 	TPDVDYLISAPVSFPLIGLVQLAHYEVTCKVLGVHPGMLRERITGSTGHSQGIVMAAATA
AN9408.1  	QPDVDYLVSAPVSLPLIGLVQLAHFAVTCRVLGKEPGEILERFSGTTGHSQGIVTAAAIA
          	 **:***:*****:**********: ***:***  *. : **::*:******:* :*  *

MG04118.1 	AADTWKEWPTIVKKTLTILFWIGARSQQTFPRTSITPSILQDSVDNGEGIPTPMLSIRDL
NCU07307.1	AADSWESFEEHSKAALTILFWIGARSQQTFPRTSLAPSMLQDSADNGEGVPTPMLSIRDL
FG05322.1 	AADSWDSWQDISSSVLTMLFWIGTRSQQAFPTTSMTPTMLRESSEHGEGSPTPMLSIRDL
AN9408.1  	TATTWESFHKAVANALTMLFWIGLRSQQAYPRTSIAPSVLQDSIENGEGTPTPMLSIRDL
          	:* :*..:      .**:***** ****::* **::*::*::* ::*** **********

MG04118.1 	PQAEVQKHIDKTNEYLPADRHISISLINSPRNMVVTGPPISLYGLNLQLRKVKAPTGLDQ
NCU07307.1	PQAEVQKHIDQTNAYLPEDQHISIALINSPRNMVVAGPPSSLHGLNLQLRKLKAATGLDQ
FG05322.1 	PQAEVQKHIDATNQYLPEDRHISISLINSPRNLVVTGPPTSLYGLNTQLRKVKAPVGLDQ
AN9408.1  	PRTAVQEHIDMTNQHLPEDRHISISLVNSARNFVVTGPPLSLYGLNLRLRKVKAPTGLDQ
          	*:: **:*** ** :** *:****:*:**.**:**:*** **:*** :***:**..****

MG04118.1 	NRIPYTQRKVRFVNRFLPITAPFHSKYLAEATSMIDNDLQYTHFEVKDLKIPVFDTHTGK
NCU07307.1	TRIPHTERKVRFVNRFLPITAPFHSKYLAKASDLIAEDLKDVQIDSRTLGMPVYNTFTGK
FG05322.1 	NRIPFTERKVRFANRFLPITAPFHSKYLSEATAMIDEDLKDINIDSSDLGIAVFDTNTGK
AN9408.1  	NRVPFTQRKVRFVNRFLPITAPFHSQYLYSAFDRIMEDLEDVEISPKSLTIPVYGTKTGD
          	.*:*.*:*****.************:** .*   * :**:  .:.   * :.*:.* **.

MG04118.1 	DIREEVKGNIVPTLIRLITRDPVHWEKATVFPGATHVLDFGPGGISGLGILTSRNKDGTG
NCU07307.1	DLREEVEGNIVPSLIRMITGETVHWEKATVFQDATHILDFGPGGISGLGILTSRNKEGTG
FG05322.1 	DLREEVKGNIIPALVRLITRDPVNWEKATVFPDATHILDFGPGGVSGLGVLTSRNKEGTG
AN9408.1  	DLRAISDANVVPALVRMITHDPVNWEQTTAFPNATHIVDFGPGGISGLGVLTNRNKDGTG
          	*:*   ..*::*:*:*:** :.*:**::*.* .***::******:****:**.***:***

MG04118.1 	VRVILAGSVAGTVTEVGYKPELFDRDEEHAVKYAIDWVKEYGPKLIKTATGRTYVDTKMS
NCU07307.1	VRVILAGTVNGTVTDVGYKPELFDRDEEQAVKFAVNWVKEYGPRLVKNSVGRTYVDTKMS
FG05322.1 	VRVILAGTVDGGMNDLGYKAELFDRDEENAVKYAIDWVKEFGPKLVNNKSGRTYLDTKMS
AN9408.1  	VRVILAGSMDGTNAEVGYKPELFDRDE-HSVKYAIDWVKEYGPRLVKNATGQTFVDTKMS
          	*******:: *   ::***.******* ::**:*::****:**:*::.  *:*::*****

MG04118.1 	RLLGLPPVMVAGMTPCTVKWDFVAATMNAGYHIELAGGGYFEPRMMTDALVKIEKAIPAG
NCU07307.1	RLLGLPPLLVAGMTPTTVPWDFIAATMNAGYQIELAGGGYYNAKTMTEAISKIEKAVPAG
FG05322.1 	RLLGLPPVLVAGMTPCTVPWDFVAATMNAGYHIELAGGGYFVGPMMTDAITKIEKAIPAG
AN9408.1  	RLLGIPPIMVAGMTPTTVPWDFVAATMNAGYHIELAGGGYYNAKTMTEAITKIEKAIPPG
          	****:**::****** ** ***:********:********:    **:*: *****:*.*

MG04118.1 	RGISVNLIYVNPRAMQWQIPLIGRLRSQGVPIEGLTIGAGVPSIEVANEYIQTLGLKHIS
NCU07307.1	RGICVNLIYVNPRAMGWQIPLLGRLRAEGYPIEGLTIGAGVPSIEVAQEYIETLGLKHIS
FG05322.1 	RGISINLIYVNPRAMAWQIPLIQKLRSEGVPIEGLTIGAGVPSIEVAQEYIETLGLKHIS
AN9408.1  	RGITVNLIYVNPRAMGWQIPLIGKLRADGVPIEGLTIGAGVPSIEVANEYIETLGIKHIA
          	*** :********** *****: :**::* *****************:***:***:***:

MG04118.1 	FKPGSTDAIQAVINIANANPTFPVMLQWTGGRGGGHHSFEDFHQPVLLMYNRIRKCDNLI
NCU07307.1	FKPGSGEAIQAVINIAKANPTFPVILQWTGGRGGGHHSFEDFHHPILNLYGRIRRQENII
FG05322.1 	FKPGSLDAIQSVINIAKANPHFPVMLQWTGGRGGGHHSFEDFHHPILQMYGRIRRQENII
AN9408.1  	FKPGSVDAIQQVINIAKANPKFPVILQWTGGRGGGHHSFEDFHQPILQMYSRIRRHENII
          	***** :*** *****:*** ***:******************:*:* :*.***: :*:*

MG04118.1 	LIAGSGFGGAEDTYPYITGEWSRKFGYPPMPFDGCLFGSRVMVAKEAHTAPAAKQAIVDA
NCU07307.1	LVAGSGFGGADDTYPYLTGDWSIKYGYPPMPFDGCMFGSRMMVAKEAHTSLAAKQAIVDA
FG05322.1 	LVAGSGFGGADDTYPYITGDWSKKYGYPPMPFDGCLFGSRMMVAKEAHTSKAAKEAIVAA
AN9408.1  	LVAGSGFGGAEDTYPYLSGNWSSRFGYPPMPFDGCLFGSRMMTAKEAHTSKNAKQAIVDA
          	*:********:*****::*:** ::**********:****:*.******:  **:*** *

MG04118.1 	PGVDDDEWEKTYNGVAGGITTVLSEMGEPIHKIATRGVKFWSEMDQKIFSLPKEKRVPEL
NCU07307.1	PGLDDSDWEKTYKGPAGGVITVRSEMGEPIHKLATRGVRFWAEMDQKIFSLPKEKRVAAL
FG05322.1 	PGLEDSEWEQTYKGPAGGVLTVRSEMGEPIHKLATRGVRFWAEMDQKIFSLPKEKRVAEL
AN9408.1  	PGLDDQDWEKTYKGAAGGVVTVLSEMGEPIHKLATRGVLFWHEMDQKIFKLDKAKRVPEL
          	**::*.:**:**:* ***: ** *********:***** ** *******.* * ***. *

MG04118.1 	KKMRSYIIEKLNKDFHKVWFGKNKAGQAVDLEDMTYAEVVRRMVELMYVKHESRWIDPSF
NCU07307.1	KASRDYIIEKLNKDFQKVWFGQNKEGKAVDLEDMTYAEIVRRLVELLYVKDEARWIDQSY
FG05322.1 	KKNKDYYIKKLNDDCQKVWFGRNKEGKAVDLDDMTYAEVLRRLVELLYVKHQSRWIDRSY
AN9408.1  	KKQRDYIIKKLNDDFQKVWFGRNSAGETVDLEDMTYAEVVHRMVDLMYVKHEGRWIDDSL
          	*  :.* *:***.* :*****:*. *::***:******:::*:*:*:***.:.**** * 

MG04118.1 	MRLTGDFIHRVEERFVQSSGQPSLLQHYTDLTQPFETIEKILAHYPQAEDQLINAQDVQH
NCU07307.1	TKLTGDFIHRIEERFTTTPGQPSRLQSYADLKEPYSAVEQILSHYPEAEKQLINAQDVQH
FG05322.1 	TVLVGDYIHRLEERLTATPGQASLLQSYSELSDPFEVIDRILAAYPDAETQIINAQDVQY
AN9408.1  	KKLTGDFIRRVEERFTTAEGQASLLQNYSELNVPYPAVDNILAAYPEAATQLINAQDVQH
          	  *.**:*:*:***:. : **.* ** *::*. *: .::.**: **:*  *:*******:

MG04118.1 	FLQLCMRPIQKPVVFVPALDDNFDFYFKKDSLWQSEDLQAVMDQDVQRTCILQGPMAVRY
NCU07307.1	FLLLCQRRGQKPVTFVPVLDENFEFFFKKDSLWQSEDLAAVVGKDVGRTCILQGPMAAKH
FG05322.1 	FLIMCMRPTQKPVTFIPVFDDNFEFYFKKDSLWQSEDLDAVVDQDVGRTCILQGPAAAKF
AN9408.1  	FLLLCQRRGQKPVPFVPSLDENFEYWFKKDSLWQSEDLEAVVGQDVGRTCILQGPMAAKF
          	** :* *  **** *:* :*:**:::************ **:.:** ******** *.:.

MG04118.1 	STVVDEPIQSILDGVHEGHIAGLSKDLYQGKTSTLPSIEYFGGKLMEREFPDVDSIDGLS
NCU07307.1	ATKVDEPIKDILDGIHNGHIALLTRDMYGGDESKIPTIEYFGGKLIESEIP--LDIEGLT
FG05322.1 	STEIDQPIKSILDDIHETHVKYLTRDQYNDNAASIPYIEYFGGKLVDPEIP--LDVEGLT
AN9408.1  	STVIDEPVGDILNSIHQGHIKSLIKDMYNGDETTIPITEYFGGRLSEAQED--IEMDGLT
          	:* :*:*: .**:.:*: *:  * :* * .. :.:*  *****:* : :     .::**:

MG04118.1 	VSEERGVRDIFRISLSPSQTMPSTDHWLSLIAGPTRSWRHALLMSEVIVQGTKFQSNPMR
NCU07307.1	VSYD-EHKNTYRLSSSPSVQLPSLDAWLSLLAGHQRNWRFALLQSDVLVQGQKYQTNPMR
FG05322.1 	VSYD-TYKNTYRLSSSAAATLPELDSWLSLLAGPNRNWRHALLMADVVAQGQKFQTNPIR
AN9408.1  	ISED-ANKISYRLSSS-AADLPEVNRWCRLLAGRSYSWRHALFSADVFVQGHRFQTNPLK
          	:* :   :  :*:* * :  :*. : *  *:**   .**.**: ::*..** ::*:**::

MG04118.1 	RIFAPCRGLFVEVRYPKDPASTQIIVREQPRHNHYVDVLVARLSGKNEIIVELIKDTTAL
NCU07307.1	RIFAPARGLFVEILHPNNPEKTVILVKEQPRPNRYAEVIEVKLDGKNEIVVNMIKDTTAL
FG05322.1 	RIFAPSRGLFVEISYPNDPSKTTIVVREQPRHNQYVDVIEVKLVGENKIQVNMIKETTAL
AN9408.1  	RVLAPSTGMYVEIANPEDAPKTVISVREPYQSGKLVKTVDIKLNEKGQIALTLYEGRTAE
          	*::**. *::**:  *::. .* * *:*  : .: ...:  :*  :.:* : : :  ** 

MG04118.1 	GKPVSLPLKFIYKPEAGYAPLHEVMDDRNDRIKEFYWRAWFGDEKLELDANVDSAFDGGK
NCU07307.1	GKPVPLTLKFIYNPEAGYAPIHEVMDGRNDRIKEFYWRAWFGDEKLDLDSSVHEVFDGGK
FG05322.1 	GKPAALPLEFTYHPEAGYSPIREVMEGRNDRIKEFYWKAWFGEEALDLDADVTAKFDGGK
AN9408.1  	NGVVPLTFLFTYHPDTGYAPIREVMDSRNDRIKEFYYRIWFGNKDVPFDTPTTATFNGGR
          	.  ..*.: * *:*::**:*::***:.*********:: ***:: : :*: .   *:**:

MG04118.1 	DVVTSEAINDFVHAVGNTGEAFVDRPGKVMYAPMDFAIVVGWKAITKPIFPRKIDGDLLN
NCU07307.1	TTITSEAINEFVHAVGNTGEAFVDRPDKVMYAPMDFAIVVGWKAITKPIFPRTIDGDLLK
FG05322.1 	TTITGEAINDFVHAVGNTGEAFVDRPGKTVYAPMDFAIVIGWKAITKPIFPRTIDGDLLK
AN9408.1  	ETITSQAVADFVHAVGNTGEAFVERPGKEVFAPMDFAIVAGWKAITKPIFPRTIDGDLLK
          	 .:*.:*: :*************:**.* ::******** ************.******:

MG04118.1 	LVHLSNQFRMKPGAEPLKKGDEVSTVSRVNAVVIQESGKMVEVCGTITRDGEPVMEVTSQ
NCU07307.1	LVHLSNQFRMMPGAEPLKKGDVVSTTAQINAVINQESGKMVEVCGTITRDGEKVMEVTSQ
FG05322.1 	LVHLSNQFRMIPGAEPLKKGDEVSTTAQINAVINQEAGKMVEVCGTLVRDGKPVMEVTSQ
AN9408.1  	LVHLSNGFKMVPGAQPLKVGDVLDTTAQINSIINEESGKIVEVCGTIRRDGKPIMHVTSQ
          	****** *:* ***:*** ** :.*.:::*::: :*:**:******: ***: :*.****

MG04118.1 	FLYRGTYTDFENTFQSKIETPMQIHLATSKDVAVLRSKEWFTLEDNVN--MDLLGQTLIF
NCU07307.1	FLYRGTYTDFENTFQRKVETPMQLHLATSKDVAVLRSKDWFMMDDVENPDIELLGQTLTF
FG05322.1 	FLYRGTYTDYENTFQRKVETPIQVHLATTRDVAILRSKDWFSVDELPQN-IELLGQTLTF
AN9408.1  	FLYRGAYTDFENTFQRKDEVPMQVHLASSRDVAILRSKEWFRLD---MDDVELLGQTLTF
          	*****:***:***** * *.*:*:***:::***:****:** ::     .::****** *

MG04118.1 	RLQSLVRFKNTTVFSSVQTRGEVLVELPTKEIVQVGSVEYEAGTSHGNPVIDFLERNGSS
NCU07307.1	RLQTLVRFKNKTVFSHVETQGQVLLELPTKEIIQVATVNYYAGESHGNPVIDYLQRHGSS
FG05322.1 	RLQSLVRYKNKTVFSSIETRGQVLLELPTKEIIQVGSVDYETGESHGNPVIDYLERNGQP
AN9408.1  	RLQSLIRFKNKNVFSQVQTMGQVLLELPTKEVIQVASVDYEAGTSHGNPVIDYLQRNGTS
          	***:*:*:**..*** ::* *:**:******::**.:*:* :* ********:*:*:* .

MG04118.1 	IEQPINFENPIPLSGKTPLQLRAPASNETYARVSGDYNPIHVSRVFANYADLPGTITHGM
NCU07307.1	IEQPINFENPIPLSGKTPLQLKAPSSNETYARVSGDYNPIHVSRVFSSYANLPGTITHGM
FG05322.1 	IDQPINFENAIPLSGRSPLALRAPASNENYARVSGDYNPIHVSRVFSSYANLPGTITHGM
AN9408.1  	IEQPVYFENPIPLSGKTPLVLRAPASNETYARVSGDYNPIHVSRVFSSYANLPGTITHGM
          	*:**: ***.*****::** *:**:***.*****************:.**:*********

MG04118.1 	YSSAAVRSLVETWAAENDNSRVRSFHASLVGMVLPNDDLLVKLQHVGMVAGRKIIKVEVI
NCU07307.1	YSSAAVRSLVETWAAENKVGRVRSFHASLTGMVLPNDDINVKLQHVGMVAGRKIIKVEAS
FG05322.1 	YSSASVRSLVETWAAENNIGRVRSFHASLVGMVLPNDDLEVKLQHSGMVSGRKIIKVEVR
AN9408.1  	YTSAAVRSLVETWAAENNIGRVRGFHVSLVDMVLPNDLITVRLQHVGMIAGRKIIKVEAS
          	*:**:************. .***.**.**..****** : *:*** **::********. 

MG04118.1 	NKETEDKVLLGEAEVEQPVTAYVFTGQGSQEQGMGMELYESSPVAKEVWDRADKYLMDTY
NCU07307.1	NKETEEKVLLGEAEIEQPITAYIFTGQGSQEQGMGMDLYNSSPVAKAVWDRADKYLMDTY
FG05322.1 	NKETEDKVLEGEAEVEQPATAYVFTGQGSQEQGMGMELYASSPVAKEVWDRADKYLLDAY
AN9408.1  	NKETEDKVLLGEAEVEQPVTAYVFTGQGSQEQGMGMELYATSPVAKEVWDRADRHFIENY
          	*****:*** ****:*** ***:*************:** :***** ******::::: *

MG04118.1 	G1FAITNIVKNNPKELTIHFGGPRGKAIRANYMAMTFETVAADGTIKSERIFKDINERTT
NCU07307.1	G1FAITNIVRNNPKELTIHFGGPRGKAIRQNYMSMTFETVAADGSIKSERMFKEIDEKTT
FG05322.1 	G1FSITNIVKNNPKELTIHFGGPRGKAIRQNYIAMTFETVAADGSIKSEKIFKEIDETTT
AN9408.1  	G~LSIIDIVKNNPKELTVHFGGPRGKAIRQNYMSMTFETVNADGTIKSEKIFKEIDETTT
          	* ::* :**:*******:*********** **::****** ***:****::**:*:* **

MG04118.1 	SYTYRSPTGLLSATQFTQPALTLMEKASFEDMKAKGLVPRDSTFAGHSLGEYSALAALAD
NCU07307.1	SYTYRSPNGLLSATQFTQPALTLMEKASFEDMKAKGLVPRDSTFAGHSLGEYSALAALAD
FG05322.1 	SYTYRSPTGLLSATQFTQPALTLMEKASFEDMKAKGLVPNSSTFAGHSLGEYSALAALAE
AN9408.1  	SYTYRSPTGLLSATQFTQPALTLMEKASFEDMRSKGLVQRDSSFAGHSLGEYSALAALAD
          	*******.************************::**** ..*:****************:

MG04118.1 	VMPIESLVSVVFYRGLTMQVAVERDASGRSNYSMCAVNPSRISKTFNEEALRFVVGNIAE
NCU07307.1	VMPIESLVSVVFYRGLTMQVAVERDETGRSNYGMCAVNPSRINKTFTEEALRFVVGSIAE
FG05322.1 	VMPIESLVSVVFYRGLTMQVAVERDAAGRSNYSMCAVNPSRISKTFNEAALQFVVDNIAE
AN9408.1  	VMPIESLVSVVFYRGLTMQVAVERDEQGRSNYSMCAVNPSRISKTFNEQALQYVVGNISE
          	*************************  *****.*********.***.* **::**..*:*

MG04118.1 	GTGWLLEIVNYNIANMQYVCAGD~LRALDTLTNVLNYLKAQKIDIEAMKAAMTVEDVKQH
NCU07307.1	TTGWLLEIVNFNVENMQYVCAGD~LRALDTLGGVCNAIKMMKIDIEKMRKEYSAETVKEQ
FG05322.1 	ETGWLLEIVNYNIANQQYVCAGD~LRALDTLAGVTNFIKIQKIDIEEVKDN--IEEVKGH
AN9408.1  	QTGWLLEIVNYNVANMQYVAAGD0LRALDCLTNLLNYLKAQNIDIPALMQSMSLEDVKAH
          	 *********:*: * ***.*** ***** * .: * :*  :***  :    : * ** :

MG04118.1 	LQEIIKASAEKTEAKPKPLELERGFATIPLRGIDVPFHSTFLRSGVKPFRSFLLKKINKT
NCU07307.1	LVEIIKSCANDTQAKPTPLVLQRGFATIPLPGIDVPFHSTFLRSGVKPFRSFLLKKINKT
FG05322.1 	LREIIRGCAEKTLAKPTPLELERGFATIPLRGIDVPFHSTFLRSGVKPFRSFLLKKINKT
AN9408.1  	LVNIIHECVKQTEAKPKPINLERGFATIPLKGIDVPFHSTFLRSGVKPFRSFLLKKINKT
          	* :**: ..:.* ***.*: *:******** *****************************

MG04118.1 	TIDPSKLLGKYIPNVTAKPFALTKEYFEDVYRLTNSPRIASILANWEQYQNENAVPSTIG
NCU07307.1	TIDPSKLIGKYIPNVTAKPFAITKEYFEDVYRLTNSPKIQQILANWDKYQEE--------
FG05322.1 	SIDPSKLVGKYIPNVTAKPFALTKEYFEDVYKLTNSPKIGAVLANWDKYNQD-------G
AN9408.1  	TIDPSKLVGKYIPNVTARPFEITKEYFEDVYRLTNSPRIAHILANWEKYEEG--------
          	:******:*********:** :*********:*****:*  :****::*::         

MG04118.1 	LGANGAGDANGETTENGVEEANGANGVH
NCU07307.1	-GVSGIPDAE------------------
FG05322.1 	VAANGVEGSE---SSSGEYEGSGRAA--
AN9408.1  	-TEGGSRHGGTTAASS------------
          	   .*   .   ::..
```
